# The Risk of Asthma in Patients with Ankylosing Spondylitis: A Population-Based Cohort Study

**DOI:** 10.1371/journal.pone.0116608

**Published:** 2015-02-06

**Authors:** Te-Chun Shen, Cheng-Li Lin, Chang-Ching Wei, Chia-Hung Chen, Chih-Yen Tu, Te-Chun Hsia, Chuen-Ming Shih, Wu-Huei Hsu, Fung-Chang Sung

**Affiliations:** 1 Graduate Institute of Clinical Medicine Science, College of Medicine, China Medical University, Taichung, Taiwan; 2 Division of Pulmonary and Critical Care Medicine, Department of Internal Medicine, China Medical University Hospital, China Medical University, Taichung, Taiwan; 3 Management Office for Health Data, China Medical University Hospital, China Medical University, Taichung, Taiwan; 4 Division of Nephrology, Department of Pediatrics, China Medical University Hospital, China Medical University, Taichung, Taiwan; Oregon Health &amp; Science University, UNITED STATES

## Abstract

**Background:**

The relationship between asthma and ankylosing spondylitis (AS) is controversial. We examined the risk of asthma among AS patients in a nationwide population.

**Methods:**

We conducted a retrospective cohort study using data from the National Health Insurance (NHI) system of Taiwan. The cohort included 5,974 patients newly diagnosed with AS from 2000 to 2010. The date of diagnosis was defined as the index date. A 4-fold of general population without AS was randomly selected frequency matched by age, gender and the index year. The occurrence and hazard ratio (HR) of asthma were estimated by the end of 2011.

**Results:**

The overall incidence of asthma was 1.74 folds greater in the AS cohort than in the non-AS cohort (8.26 versus 4.74 per 1000 person-years) with a multivariable Cox method measured adjusted HR of 1.54 (95% confidence interval (CI), 1.34–1.76). The adjusted HR of asthma associated with AS was higher in women (1.59; 95% CI, 1.33–1.90), those aged 50–64 years (1.66; 95% CI, 1.31–2.09), or those without comorbidities (1.82; 95% CI, 1.54–2.13).

**Conclusion:**

Patients with AS are at a higher risk of developing asthma than the general population, regardless of gender and age. The pathophysiology needs further investigation.

## Introduction

Spondyloarthropathies (SpAs) are a group of interrelated diseases characterized by joint inflammation and extra-articular involvement, including mainly the axial and peripheral type and extra-articular type [1–3]. Ankylosing spondylitis (AS) is an axial and peripheral type of SpA, which mainly affects the joints of the axial skeleton; however, extra-articular features may also occur. AS can affect the tracheobronchial tree and pulmonary parenchyma and is associated with several pulmonary manifestations [4–12]. Recent studies reported in the United States that the overall age-adjusted prevalence of SpA ranged from 0.9%–1.4% based on the diagnosis criteria, with the prevalence rates similar in men and women [12, 13]. Another epidemiologic study has reported a prevalence of 16.7 per 10,000 in Asian population [14].

Asthma is a chronic inflammatory disorder of the airways and it is a representative disease of atopic disorders. Rudwaleit *et al* have reported that the prevalence rates of atopic disorders were 61/248 (24.6%) in AS patients and 111/536 (20.7%) in the control population, while the prevalence rates of asthma were 18/248 (7.3%) in AS patients and 35/536 (6.5%) in the control population. They concluded that there was a slight but insignificant increase in atopic disorders in AS patients [15]. However, this study used a questionnaire-based survey involving a small study population. In two nationwide population-based cohort studies of ours, an increased risk of asthma was identified in patients with systemic lupus erythematosus (SLE) and rheumatoid arthritis (RA) [16–17]. AS is thought to have an autoimmune component, we suggest that AS may be associated with asthma occurrence.

Autoimmune diseases (ADs) are caused by the loss of immune tolerance and are characterized by T- or B-cell activation leading to tissue damage. Patients with AD can be defined by a direct and indirect circumstantial evidence. Because of lack of the direct proof, patients with AS have limited features of AD [18]. However, patients with AS also exhibits the features of autoinflammation with activated innate immune system, which may appear at specific disease-prone sites and be determined by local factors [19]. However, a definitive relationship between AS and asthma remains unclear.

This study attempts to determine whether patients with AS are at an elevated risk of developing asthma. We conducted a retrospective cohort study using nationwide population-based data, the National Health Insurance Research Database (NHIRD) of Taiwan.

## Materials and Methods

### Data Source

The National Health Insurance (NHI) program in Taiwan was launched in 1995 to provide comprehensive medical care for over 99% of the 23.74 million people residing in Taiwan (http://www.nhi.gov.tw/english/index.aspx). The NHIRD, established by the Bureau of National Health Insurance (BNHI) and the National Health Research Institutes (NHRI), is one of the large administrative health care databases in the world and is available to scientists for research purposes. All identification numbers of insured people and healthcare facilities in this database are encrypted for privacy protection. The encrypted identification numbers are unique, enabling record linking. The present study used the electronic data of the Longitudinal Health Insurance Database (LHID) obtained from NHRI, consisting of claims data of 1,000,000 individuals randomly sampled from all population. The data can be retrospectively traced from 1996 onwards and followed up to the end of 2011. The NHRI reported no statistically significant differences in gender, age, and health care cost distribution between these subjects and whole insured population. This study was exempted from full ethical review by the China Medical University and Hospital Research Ethics Committee (IRB permit number: CMU-REC-101-012). The diagnostic code is in the format of the International Classification of Disease, 9th Revision, Clinical Modification (ICD-9-CM).

### Sampled Participants

We identified a cohort of patients newly diagnosed with AS (ICD-9-CM code: 720 and 720.0) [20, 21] between January 1, 2000 and December 31, 2010. The date of the initial diagnosis was defined as the index date. Patients with a history of asthma (ICD-9-CM code: 493) or those with missing age or gender information were excluded. For each AS case identified, four insured controls adhering to the same inclusion criteria were randomly selected into the non-AS cohort. The subjects were frequency-matched by age (within 5 year), gender, and index year to reduce confounding effects of age and gender and to reduce measure bias of follow-up.

### Outcome and relevant variables

Each subject was monitored from the index date until a new diagnosis of asthma was reached or until the subject was eliminated because of failure of follow-up, death, withdrawal from the insurance system, or the end of follow-up on December 31, 2011. The baseline history of comorbidity and medication for each subject was determined from the claims data, including rhinitis (ICD-9-CM codes: 472.0 and 477), chronic sinusitis (ICD-9-CM code: 473), gastroesophageal reflux disease (GERD; ICD-9-CM codes: 530.11, 530.81), obstructive sleep apnea (OSA; ICD-9-CM codes: 327.23, 780.51, 780.53, 780.57), chronic obstructive pulmonary disease (COPD; ICD-9-CM codes: 496) and usage of aspirin and non-steroidal anti-inflammatory drugs (NSAIDs) [22, 23].

### Statistical analysis

Distributions of categorical demographic factors including gender and age (<20, 20–34, 35–49, 50–64 and ≥65 years) and comorbidities including rhinitis, chronic sinusitis, GERD, OSA, COPD, and usage of aspirin and NSAIDs, were compared between the AS and non-AS cohorts. Differences were examined using the Chi-square test for categorical variables and the *t*-test for continuous variables. The incidence density rates by gender, age, and comorbidity for each cohort were estimated by follow-up time (in person-years). The AS cohort to non-AS cohort incidence rate ratio (IRR) with 95% confidence intervals (CI) was determined using the Poisson regression model. The multivariable Cox proportional hazards regression model was used to assess the risk of asthma associated with AS, comparing with the non-AS cohort and adjusted for demographic factors, comorbidities of rhinitis, chronic sinusitis, GERD, OSA, COPD, and usage of aspirin and NSAIDs. We also used the Kaplan–Meier method to estimate the cumulative asthma incidence for the two cohorts in the follow-up period and examined the difference using the log-rank test. All analyses were performed using the SAS statistical package (version 9.3 for windows; SAS Institute, Cary, NC), with the statistical level of significance being 0.05.

## Results

**Fig 1:**
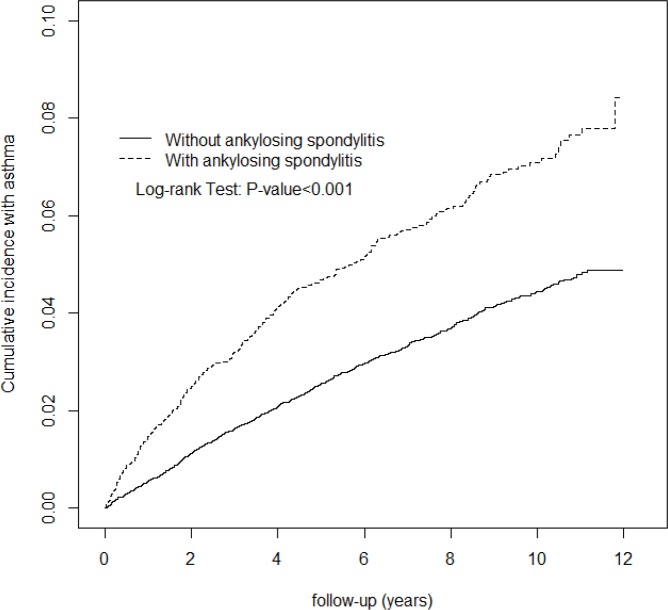
Cumulative incidence of asthma in patients with and without ankylosing spondylitis.

The study cohorts consisted of 5,974 AS patients and 23,896 non-AS control subjects. The age and gender distributions were similar in both cohorts (Table 1). Most subjects were 20–49 years of age (53.7%) in both cohorts. Mean ages of the AS and non-AS cohorts were 46.3 ± 17.4 and 45.9 ± 17.7 years, respectively. Comorbidities including rhinitis, chronic sinusitis and GERD, and usage of aspirin and NSAIDs were more prevalent in AS patients than controls (p-values, <0.001). The average follow-up period was 6.67 ± 3.30 years (39,838 person-years) for the AS cohort and 6.72 ± 3.27 years (160,617 person-years) for the non-AS cohort. The Kaplan–Meier analysis showed that by the end of the 12-year follow-up period, the cumulative probability of developing asthma was 3.7% higher for the AS cohort than the non-AS cohort (log-rank test, p < 0.001; Fig. 1).

**Table 1:** Demographic characteristics, comorbidity and medication in patients with and without ankylosing spondylitis.

Variables	Ankylosing spondylitis				p-value^†^
	No (N = 23,896)		Yes (N = 5,974)		
	n	%	n	%	
Sex					0.99
Male	12620	52.8	3155	52.8	
Female	11276	47.2	2819	47.2	
Age, years					0.99
<20	1233	5.16	308	5.16	
20–34	5939	24.9	1485	24.9	
35–49	6888	28.8	1722	28.8	
50–64	5884	24.6	1471	24.6	
≥65	3952	16.5	988	16.5	
Mean (SD)^†^	45.9	17.7	46.3	17.4	0.13
Comorbidity					
Rhinitis	2938	12.3	1052	17.6	<0.001
Chronic sinusitis	381	1.59	152	2.71	<0.001
GERD	218	0.91	129	2.16	<0.001
OSA	56	0.23	34	0.57	<0.001
COPD	1294	5.42	449	7.52	<0.001
Medication					
Aspirin	4795	(20.1)	1493	(25.0)	<0.001
NSAIDs	8929	(37.4)	4017	(67.2)	<0.001

Chi-square test

^†^Two sample t-test

GERD, gastroesophageal reflux disease; OSA, obstructive sleep apnea; COPD, chronic obstructive pulmonary disease; NSAIDs non-steroidal anti-inflammatory drugs.

Table 2 shows there were a total of 1,090 asthma events. The incidence was 1.74-fold (95% CI = 1.53–1.98) higher in the AS cohort than in the non-AS cohort (8.26 vs. 4.74 per 1000 person-years), with an adjusted hazard ratio (HR) of 1.54 (95% CI = 1.34–1.76) after controlling for age, gender, comorbidities and medications. The incidence of asthma was higher in women than in men in both cohorts. The adjusted HR of asthma in men and women was 1.47 (95% CI = 1.20–1.80) and 1.59 (95% CI = 1.33–1.90), respectively. The incidence increased with age in both cohorts. The age-specific adjusted HRs for the AS cohort, compared with the non-AS cohort, were significant for all age groups, except the youngest age group. Among those without comorbidities, the AS patients had an even higher adjusted HR of 1.82 (95% CI = 1.54–2.13) for asthma. Table 3 shows that comorbidities increased the adjusted HR of asthma to 2.49 (95% CI = 1.99–3.12) for the AS cohort.

**Table 2:** Incidence of asthma and Cox method estimated ankylosing spondylitis cohort to non-ankylosing spondylitis cohort hazard ratio.

Variables	Ankylosing spondylitis						Crude HR^§^ (95%CI)	Adjusted HR^†^ (95%CI)
	No (N = 23,896)			Yes (N = 5,974)				
	n	Person-years	rate^#^	n	Person-years	rate^#^		
Total	761	160617	4.74	329	39838	8.26	1.74(1.53,1.98)***	1.54(1.34, 1.76)***
Sex								
Male	341	84939	4.01	141	21186	6.66	1.66(1.36,2.02)***	1.47(1.20,1.80)***
Female	420	75677	5.55	188	18652	10.1	1.81(1.53,2.15)***	1.59(1.33,1.90)***
Age, years								
<34	131	48936	2.68	51	12468	4.09	1.53(1.11,2.12)***	1.24(0.88, 1.75)
35–49	176	49246	3.57	86	12194	7.05	1.97(1.52,2.55)***	1.55(1.18, 2.03)***
50–64	237	39881	5.94	108	9651	11.2	1.88(1.50,2.36)***	1.66(1.31, 2.09)***
≥65	217	22553	9.62	84	5524	15.2	1.57(1.22,2.03)***	1.51(1.17, 1.95)**
Comorbidity								
None^‡^	517	137393	3.76	233	31558	7.38	1.97(1.69,2.30)***	1.82(1.54,2.13)***
Rhinitis	128	16397	7.81	55	5898	9.33	1.19(0.87, 1.64)	1.17(0.85, 1.60)
Chronic sinusitis	20	2179	9.18	15	869	17.3	1.84(0.94, 3.60)	1.79(0.91, 3.51)
GERD	3	732	4.10	4	423	9.47	2.20(0.49, 9.84)	2.39(0.52, 10.9)
OSA	0	232	0.00	0	142	0.00	-	-
COPD	143	7172	19.9	44	2622	17.8	0.85(0.61, 1.19)	0.86(0.61, 1.21)
Medication								
Aspirin								
No	565	132646	4.26	242	30937	7.82	1.84(1.58,2.14)***	1.62(1.39, 1.89)***
Yes	196	27971	7.01	87	8902	9.77	1.40(1.09,1.80)**	1.32(1.02, 1.71)*
NSAIDs								
No	389	108914	3.57	89	14935	5.96	1.68(1.33,2.11)***	1.60(1.27, 2.02)***
Yes	372	51703	7.19	240	24903	9.64	1.36(1.16,1.60)***	1.48(1.26, 1.74)***

Rate^#^ per 1000 person-years

Crude HR^§^, relative hazard ratio

^†^ Model was adjusted for age and comorbidities of rhinitis, chronic sinusitis, GERD, OSA, COPD and usage of aspirin and NSAIDs

^‡^ Patients without any one of the comorbidities (rhinitis, chronic sinusitis, GERD, OSA, and COPD) were classified as the none group

*p < 0.05, **p < 0.01, ***p < 0.001

**Table 3:** Cox method estimated hazard ratios of asthma associated ankylosing spondylitis and comorbidity.

Variables		Event		Adjusted HR^†^ (95%CI)	p-value^#^
Ankylosing spondylitis	Comorbidity^‡^	N	n		<0.001
No	No	19716	517	1(Reference)	
No	Yes	4180	244	2.31(1.98, 2.70)***	
Yes	No	4493	233	1.83(1.56, 2.14)***	
Yes	Yes	1481	96	2.49(1.99, 3.12)***	

^†^ Model was adjusted for age, sex and usage of aspirin and NSAIDs

^#^p-value for interaction

^‡^ Patients with any one of the comorbidities of rhinitis, chronic sinusitis, GERD, OSA, and COPD were classified as the comorbidity group

***p < 0.001

## Discussion

To the best of our knowledge, this is the first nationwide population-based study evaluating the relationship between AS and subsequent risk of asthma. We identified a significant risk of asthma among AS patients compared to the general population. Sex-specific analysis shows the highest asthmatic risk for females with AS patients. In addition, we found AS patients without comorbidities had a near 2-fold higher asthmatic risk than controls without comorbidities. Our data suggest that AS may play an independent role in the development of asthma.

The incidence of asthma increased with age in both cohorts. However, the crude AS patients to controls HR was the highest in the 30–49 years. The possible explanation for this might be that older subjects in both cohorts tend to be more prevalent with comorbidities, which may account for late-onset asthma. Risk factors may also include occupational exposure to irritants, environmental pollutants, female sex hormones, upper airway diseases, medications, respiratory infections, obesity, and stressful life events [24]. The crude HR of the older age groups was therefore lower than the younger age groups.

Few studies have discussed the relationship between AS and risk of asthma. Rudwaleit *et al*. reported the prevalence of asthma to be 7.3% in AS patients and 6.5% in a control population [15]. They used a questionnaire-based method and concluded that there is a slight and insignificant increase in asthma in AS patients. The diagnosis of asthma in AS patients is a challenge for physicians because pulmonary function tests are often influenced by chest wall restriction. Dincer et al. have reported that AS patients may experience deteriorated pulmonary function: the forced expiratory volume during the first second (FEV1) and FEV1 to forced vital capacity (FVC) ratio are decreased in AS patients as compared with controls [25]. Therefore, the diagnosis of asthma should be based on characteristic symptom patterns and evidence of variable airflow limitation from the bronchodilator reversibility or bronchial provocation tests, rather than from absolute values obtained from pulmonary function tests.

Several studies have shown a decreased risk of atopic diseases in patients with autoimmune diseases such as RA [15, 26–27]. However, these studies were primarily either only questionnaire based or using a small study population. Most researchers assumed that the establishment of an atopic phenotype reduces the risk of the subsequent development of autoimmune diseases. AS has both autoimmune component and autoinflammatory component. An early study has shown that AS is associated with HLA-B27 which may modulate the inflammatory response via misfolding with an unfolded protein response and/or via antigen recognition. Diseases associated with HLA-B27 are more likely autoinflammatory than autoimmune in nature [28]. However, the causality between AS and asthma remains largely unknown.

There are a number of factors that influence the risk of developing asthma. Although asthma is a representative atopic disorder, the pathophysiological mechanisms are very complex. Development of asthma can be attributed to host factors such as genetics, obesity, and gender and environmental factors such as allergens, infections, occupational exposure, tobacco smoke, air pollution, and diet. Additionally, racial and ethnic differences, lifestyle, socioeconomic status, and the efficacy of the public health system have been linked to the prevalence of asthma [14]. In the present study, non-atopic asthma may also play a role in AS patients. To summarize, the increased risk of asthma in AS patients does not reflect an association between atopic asthma and AS.

A major strength of our study is that it was performed using population-based data that are highly representative of the general population. However, certain limitations should be considered. First, this study used the ICD-9-CM algorithm to define AS, asthma and comorbidities. The diagnosis depends on the performance of clinical physicians. An ad hoc committee established by the insurance authority was in charge of evaluating the claims data to prevent errors and violation. In addition, we selected only diagnosis appeared at least twice within a year to increase the validity and accuracy of diagnosis. Second, information on smoking, diet, occupational exposure, and family disease history was not available in the data analysis for adjusting the association between AS and asthma. Third, relevant clinical data such as pulmonary function tests, laboratory data on urinary and blood tests, imaging results, and pathology findings of the subjects were also unavailable in our study. Therefore, the statistical quality in a retrospective cohort study may be weaker.

## Conclusion

Our data suggest that patients with AS have a significantly higher risk of developing asthma than the general population regardless of gender and age. Although, the pathophysiology association between AS and asthma needs further investigation.
